# Hydrotherapy and acupressure for restless legs syndrome: results of a qualitative part of a randomized controlled exploratory study

**DOI:** 10.3389/fmed.2025.1607307

**Published:** 2025-09-01

**Authors:** J. Siewert, J. Kubasch, B. Brinkhaus, M. Teut, E. Jansen

**Affiliations:** ^1^Institute of Social Medicine, Epidemiology and Health Economics, Charité University Medicine Berlin, Berlin, Germany; ^2^Institute of Social Medicine, Epidemiology and Health Economics, The Brandenburg Medical School Theodor Fontane, Neuruppin, Germany

**Keywords:** restless legs syndrome, qualitative research, sleep disorders, complementary and alternative medicine, mixed methods study, hydrotherapy, acupressure

## Abstract

**Introduction:**

Restless legs syndrome (RLS) has a negative impact on quality of life and remains challenging to treat. This study explored the subjective experiences of individuals using two self-managed, non-pharmacological interventions—hydrotherapy and acupressure—for RLS, focusing on their perceived benefits, challenges, and feasibility in everyday life.

**Methods:**

Within a three-armed randomized study we conducted qualitative interviews in both intervention groups. The semi-structured interviews were coded and analyzed using reflexive thematic analysis. Coding followed both an inductive approach, grounded in the data, and a deductive approach, guided by the study objectives. Data analysis was carried out using MAXQDA® software.

**Results:**

A total of 12 telephone interviews (six per intervention group) were qualitatively analyzed. Participants had a mean age of 65 years (range 35–75). Six themes emerged: prior experiences, motivation, study document perception, treatment integration, perceived impact, and post-intervention use. Both groups reported symptom and sleep improvements. Increased mindfulness was more common in the acupressure group. Hydrotherapy participants noted sensory effects but also discomfort and time barriers. Both interventions were seen as practical, with acupressure being perceived as easier to apply.

**Conclusion:**

Participants of both groups reported varying degrees of RLS symptom improvement. High-quality confirmatory RCTs are needed to investigate the effectiveness of acupressure and hydrotherapy, with a focusing on practical implementation and the need for comprehensive, continuous guidance. Treatments in RLS should be location-independent to improve both participation and outcomes. Future research should further explore individualized adaptations and contextual factors influencing treatment experience and effectiveness.

**Trial registration:**

Identifier DRKS00029960.

## Introduction

Restless legs syndrome (RLS) is a chronic, progressive, and primarily sensorimotor disorder. The prevalence of RLS differs depending on the assessment method and target group. According to a recent meta-analysis, the prevalence of restless legs syndrome in Europe is 5.9% (1). The pathophysiology of RLS is not sufficiently understood. Various factors such as iron levels, oxidative stress, dopamine metabolism, alterations of brain connectivity and blood oxygenation, appear to be involved in the pathophysiology of RLS (2–6). Investigations using multimodal MRI techniques also indicate a complex and not yet fully understood pathophysiology of RLS (7–9). The disease can lead to unpleasant sensations, often described as tingling, burning or even pain in the legs, which occur particularly in the evening and at night as well as during inactivity, resulting in an urge to move and sleep disturbances. The sleep disturbances in RLS are primarily caused by a combination of sensory symptoms and motor phenomena, most notably periodic limb movements during sleep (PLMS), which are frequently associated with the condition. Patients with RLS commonly experience difficulties initiating sleep, frequent nocturnal awakenings, reduced total sleep time and sleep efficiency, as well as non-restorative sleep, which may result in excessive daytime fatigue and impaired functioning (10, 11). As a result, there is significant impairment in everyday life and a reduction in the quality of life. RLS shows an association with depression and anxiety disorders (12–17). There is probably a bidirectional relationship between depressive symptoms and RLS (18). The RLS symptoms are more pronounced under psychological stress (18).

RLS is often treated pharmacologically. One in five patients with RLS would like to receive medical treatment (19). However, many RLS patients are dissatisfied with pharmacological treatment (20–22). Dopaminergic and the opioidergic and anticonvulsant substances are available for drug therapy (23). Nonetheless, the desired effect of these drugs does not last for many patients. The most significant side effect in up to 60% of RLS patients is augmentation (24–26). In addition to gastrointestinal complaints, other side effects include the possible occurrence of impulse control disorders (27). Children can also be affected, which poses considerable challenges for treatment, especially drug therapy (28–30). The updated clinical practice guideline of the American Academy of Sleep Medicine no longer recommends dopamine agonists such as pramipexole and ropinirole as standard therapy, issuing a conditional recommendation against their routine use due to the risk of augmentation. Instead, non-dopaminergic agents like gabapentin enacarbil, gabapentin, and pregabalin are strongly recommended as first-line treatments, while low-dose opioids (e.g., oxycodone/naloxone) are reserved for refractory cases under close monitoring (23). According to a survey conducted in 2004, up to 65% of RLS patients regularly use Complementary and Alternative Medicine (CAM) treatments to alleviate their RLS symptoms (31).

Previous studies on acupuncture (32–35) and acupressure (36) showed that these treatments for RLS were significantly superior to the control group in terms of reducing symptom severity. Most research to date has been conducted on acupuncture.

There are also indications that cold applications can cause a reduction in symptoms (32–34). In a controlled study, Kneipp hydrotherapy showed a significant improvement in sleep quality and well-being in non-organic sleep disorders (37).

There has been little qualitative research in the field of restless legs syndrome and complementary medicine. A systematic qualitative review of studies on patients’ experiences with acupuncture identified recurring themes, including anticipatory hope prior to treatment, a relaxing effect during sessions, perceived positive body–mind outcomes afterwards, and the significance of the therapeutic relationship with the practitioner (38). In a qualitative study on hydrotherapy, postmenopausal women reported, among other things, reduced pain and tension, better quality of life and psychosocial stabilization, especially through stress reduction and social interaction (39). A qualitative study on restless legs syndrome revealed that participants’ experiences were characterized by disempowerment, frustration, and the ineffectiveness of treatments. The feeling of being understood by healthcare professionals through validating communication was perceived as a key factor for empowerment (40). The HYDRAC study (Hydrotherapy according to Kneipp and acupressure for Restless Legs Syndrome) revealed that participants’ experiences were characterized by disempowerment, frustration, and the ineffectiveness of treatments. The feeling of being understood by healthcare professionals through validating communication was perceived as a key factor for empowerment (3).

The HYDRAC study (Hydrotherapy according to Kneipp and acupressure for Restless Legs Syndrome) aimed to investigate the effects and feasibility of two home-based interventions—hydrotherapy and acupressure—in individuals with RLS. In this three-arm randomized controlled trial (RCT), both interventions were evaluated in addition to routine care, compared to a control group receiving routine care only. Further details on study design, intervention procedures, and outcome measures can be found in the published study protocol (41). Quantitative findings from this exploratory pilot study suggest potential benefits of both interventions for quality of life, and of hydrotherapy in particular for symptom severity (42).

In this article we present the results of the qualitative part of the HYDRAC study. The aim of this part of the study was to investigate the impact the interventions hydrotherapy and acupressure had on the participants, the difficulties faced in integrating it into their daily lives, and how they continued to adapt the learned therapeutic approaches to their lives after the intervention.

## Methods

### Study background and participant recruitment

The qualitative analysis is based on an interview study with 12 participants from the two arms of intervention of the HYDRAC study. The inclusion criteria for the HYDRAC study were a confirmed RLS diagnosis according to IRLSSG criteria, RLS-related complaints ≥ 30 mm on Visual Analog Scale (0–100 mm), moderate to severe RLS symptoms (International Restless Legs Syndrome Study Group Rating Scale [IRLS] total score ≥ 11). The exclusion criteria were an indication for iron replacement therapy, regular intake of medications that trigger RLS, an existing opioid therapy and the use of hydrotherapy, acupuncture, or acupressure in the last 4 weeks or planned in the next 12 weeks.

### Sampling strategy and recruitment process

At baseline, participants were additionally asked whether they were willing to take part in a qualitative interview about their experiences with the intervention they received. Participants who had expressed interest were placed on a randomized list to reduce selection bias. From this list, participants were purposefully selected in that order to ensure equal representation across intervention groups. This approach combined random ordering with strategic sampling to achieve a balanced range of experiences and perspectives. It enabled diversity regarding the intervention, life circumstances, and symptom severity. Recruitment continued until six participants from each intervention group were included, providing sufficient data richness and thematic saturation.

### Timing and conducting of interviews

Participants were interviewed after completing the 12-week study intervention. All interviews were conducted by telephone by the trained physician and researcher JS due to the restrictions of Covid-19 after completion of the intervention. No prior relationship existed between the participants and the interviewer before the study began. Before the interviews, participants were informed about JS’s role as a researcher and the purpose of the study. The study was grounded in a methodological orientation of reflexive thematic analysis (43), which emphasizes the active role of the researcher in the analytic process and acknowledges how their perspectives and interactions influence interpretation.

### Data collection procedure

The qualitative interviews were conducted between September 2022 and March 2023. Once the target of 12 interviews (six per intervention group) was achieved, the recruitment phase was closed. There were no participants who refused to participate or dropped out. Interview duration ranged from 12 to 39 min, with an average length of 23.5 min.

Informed consent was obtained in writing from all participants prior to the interviews. Participants were informed about the study’s purpose, their rights—including the voluntary nature of participation and the possibility to withdraw at any time—and the procedures for data protection.

All interviews were conducted by telephone and were digitally recorded. No field notes were made during or after the interviews. Participants had no prior relationship with the interviewer.

### Interview content and guide development

The semi-structured interview guideline emphasized medical and life-related impact after implementing the intervention, motivation and expectations before the study, quality of life and well-being and life in general. Additionally, as part of the process evaluation, participants were asked about the study’s implementation, the paperwork, questionnaires and diaries involved, and general bureaucratic processes (see questionnaire in Appendix 1). The interview guideline was developed based on existing literature, expert knowledge and the research objectives.

### Data protection and transcription

The interviews were digitally recorded and stored in encrypted form in accordance with data protection regulations. After secure storage, the recordings were immediately deleted from the recording device. The audio files were securely transmitted to a professional transcription service and transcribed verbatim. During transcription, pseudonyms were assigned and any identifying information was removed. All data were transferred and stored in an encrypted and pseudonymized format. The transcripts will be retained for 10 years before deletion. Transcripts were not returned to participants for comment or correction, and no participant feedback was obtained on the findings.

### Data analysis

Data were analyzed using reflexive thematic analysis following Braun and Clarke’s six-phase approach (43): familiarization with the data, generation of initial codes, searching for themes, reviewing themes, defining and naming themes, and producing the report. Transcripts were coded by the author EJ using MAXQDA®. Coding was conducted both inductively—guided by the interview material—and deductively, based on the study objectives.

In a first step, initial codes were generated line by line. These codes were then grouped into descriptive categories that captured recurring content across interviews. In the next step, categories were iteratively compared, refined, and synthesized into overarching analytical themes, which reflected broader patterns in participants’ experiences and interpretations.

Initial coding frameworks and emerging themes were discussed within the research team to promote reflexivity and analytic depth. The two intervention groups were first analyzed separately to identify possible group-specific aspects and then compared and merged to develop cross-cutting themes. The analytic process was enriched by interdisciplinary team discussions and critical reflection throughout.

### Ethical considerations

This study was approved by the Ethics Committee of Charité—Universitätsmedizin Berlin (EA2/132/22, 12.07.2022). All participants provided written informed consent. The study adhered to the ethical principles of the Declaration of Helsinki (2013 version) and followed all applicable data protection regulations, including GDPR. Participation was voluntary, and participants could withdraw at any time without consequences. All data were treated confidentially and were pseudonymized.

## Results

The qualitative sample was heterogeneous: it included women and men of different age groups (35–75 years), with varying lengths of RLS experience (2–45 years) and diverse professional backgrounds. This diversity contributed to a wide range of perspectives on the treatment of RLS and the use of complementary methods.

At the time of the interviews, seven participants were already retired. When asked to assess the severity of their illness, two participants described themselves as seriously ill, seven as moderately ill, two as slightly ill, and one person as slightly to moderately ill.

Six participants were assigned to the hydrotherapy group and six to the acupressure group. Table 1 provides an overview of participants’ sex, age, professional background, self-reported RLS severity, and intervention group allocation.

**Table 1:** Demographic and clinical characteristics of interview participants.

ID	Sex	Age (years)	Profession	Duration of RLS (years)	Self-reported severity	Study intervention group
1	M	56	Computer scientist	15	Severe	Hydrotherapy
2	F	67	Retired (former teacher)	45	Medium	Hydrotherapy
3	M	58	Computer scientist	10	Mild to Medium	Hydrotherapy
4	M	56	Retired (former postal worker)	2	Severe	Hydrotherapy
5	M	66	Retired (former gardener)	4	Medium	Hydrotherapy
6	F	59	Retired (former agricultural economist)	40	Mild	Hydrotherapy
7	M	49	Employee	6	Medium	Acupressure
8	F	75	Retired (formerly in pharmaceutical industry)	8	Medium	Acupressure
9	F	71	Retired (former bank employee)	6	Medium	Acupressure
10	M	35	Service technician	15	Mild	Acupressure
11	F	60	Salesperson	25	Medium	Acupressure
12	F	68	Retired	40	Medium	Acupressure

### Study participants

#### Overview of the results

Thematic analysis of the interviews yielded six overarching themes: (1) prior experiences with complementary and alternative medicine (CAM), (2) motivation for study participation, (3) perception of study documentation (including clarity, comprehensibility, and administrative burden), (4) integration of the intervention into daily life, (5) impact on sleep, well-being, and symptom relief, and (6) post-intervention management.

These themes and their associated categories are summarized in Table 2. The table also indicates the number of participants (n) who referred to each category at least once during the interviews. Most themes and categories were reported across both intervention groups. Only a few subthemes showed group-specific tendencies: for example, *increased mindfulness* and *difficulties reaching the correct point* were reported only by participants in the acupressure group, while *improved sense of temperature* and *interest in additional therapy* were exclusive to the hydrotherapy group.

**Table 2:** Overview of key themes and reported experiences by intervention group.

Themes	Categories	No. of participants (*n*)
1. Prior experiences to CAM	No specific pattern	8
2. Motivation to use CAM	Symptom relief	8
	Reduction of medication	4
3. Perception of study documentation	Too few and imprecisely asked	6
	Positive for reflection	5
4. Integration of acupressure and hydrotherapy into daily life	Practical and little effort	10
	Uncertainty of correct implementation	3
	No time for implementation	3
	Difficulties reaching point	2
	Very pleasant	2
5. Impact of acupressure and hydrotherapy on RLS	Overall improvement of symptoms	5
	No symptom improvement	7
	Sleep improvement	4
	Improved sense of temperature	4
	Increased mindfulness	3
	Sense of autonomy/self-determination	2
6. Post-intervention management of acupressure and hydrotherapy	Adaptation of the intervention	5
	Interest in additional therapy	3

The following presentation of results is structured along these six main themes. To ensure narrative coherence and account for thematic overlap, the first three themes—prior experiences, motivation, and perception of study documents—are presented together in one section.

### Prior experiences, motivation and perception of study documents

During the analysis of the interviews, no differences were found in the prior experiences, motivation and study document of CAM categories from the participants’ perspective among the two groups. Therefore, the content is shared and combined together here.

The participants’ previous experiences and general attitudes toward CAM showed a wide variety and no consistent pattern. Participants have utilized a variety of non-conventional CAM therapy options, which include acupuncture, consumption of hemp, cold showers, osteopathy, meditation, insomnia therapy, squats, calf wraps, and Schüßler salts.

Motivations for participating in the study and trying CAM included hopes for symptom relief, the desire to reduce medication intake, as well as interests in social support, contributing to scientific research, and improving quality of life. Perceptions of the study documentation varied. Some participants felt that the information provided was too limited and superficial. As one person stated: “During the presentation, when I was here, I found everything very brief; I had imagined all of this to be much more elaborate overall. Maybe it’s different now, but I just found everything too superficial. Just a few questions, then the little booklet was shown, and she briefly went through it with me.” (participant 9). Others appreciated the documentation as an opportunity for self-reflection, as illustrated by the following remark: “Yes, that was fine, and yes, it does make you think a little more. So I definitely found it positive.” (participant 12). A few participants also mentioned discomfort with certain questions—especially those related to sexuality—which they felt were too personal for the study context.

### Integration of acupressure and hydrotherapy into daily life

Regarding the integration of the interventions into daily life, all six participants of the acupressure group perceived the application as very practical and requiring only little effort. One participant informed us that it is very time-efficient: “I thought it was good that it does not take too long, that you can get through it in 14 or 16 min, which I think can be easily incorporated into your routine at least once a day during your leisure time.” (participant 7). Another participant pointed out that the therapy can be carried out in any location: “I think with acupressure, you can perform it anywhere. I have done it sometimes when we drove from home to B., simply starting it during the drive, where I already pressed a few points.” (participant 10).

Uncertainty about the correct application of the method emerged as a recurring issue among participants in the acupressure group. This is illustrated by the following quote: “I sometimes feel like I might be pressing wrongly or something; am I in the right place? Or how far is the action radius, that is, how far can I be from this point now? Is it about centimeters or millimeters that I might not be hitting this point correctly?” (participant10).

Some participants also described physical challenges in reaching certain acupressure points. One person said: “Especially when you have points at the foot or something where you inevitably have to bend down and then, for example, make your back arch, it sometimes becomes uncomfortable for your back during the actual acupressure. You have to get used to it a bit, or you just end up sitting uncomfortably or in an unfavorable position because there’s no other way, but it’s not for a very long time.” (participant 7).

Additional challenges mentioned included discomfort or pain during the application, and limited time available in daily life.

Hydrotherapy was frequently described as practical and easy to integrate into daily routines.

As one person said: “What I really liked was that it can be easily integrated into the daily routine with a fairly manageable and minimal effort.” (participant 3). Some participants described the intervention as particularly pleasant, as indicated here: “It was quite pleasant. It was cold water, but since you are in a warm apartment, it wasn’t so cold that you would think, oh my goodness, this torture again. No, I mean, that’s what I mean by harmless; it was certainly bearable.” (participant 5).

However, difficulties with regular implementation and discomfort with the water temperature were also noted by several participants: “I was also glad that it was over afterwards because I did not have to think about it anymore.” (participant 6). One participant mentioned that the water is uncomfortably cold.

### Impact of the intervention on sleep, well-being, and symptom relief

However, difficulties with regular implementation and discomfort with the water temperature were also noted by several participants. One person expressed it this way:

“I have experienced that, as I said, I felt that there is certainly a positive attitude toward it, I’m doing this now, and it just has to get better. And I felt that this actually turned out to be true, at least I have the impression that it is somewhat alleviated.” (participant 8).

Others, however, expressed that they did not notice any meaningful changes: “In my opinion, I did not feel any effects. So, if I now have a general improvement in symptoms, that is certainly positive overall, but I did not perceive any concrete or significant improvement in that sense.” (participant 7).

Some participants described a heightened sense of mindfulness and attentiveness as a result of engaging with the acupressure practice: “What do I attribute this to? To the acupressure itself, perhaps also because one becomes more aware of the topic again. So you pay more attention to everything, because you are dealing with it.” (participant 10). Improved sleep and feelings of self-determination were also mentioned. One person described it this way: “Or falling asleep more easily; for me, it has always been falling asleep—once I am asleep, I do not have any more problems, and yes, falling asleep has simply worked better.” (10). Another person reported that the increase in medications was smaller than expected, one reported irregular bowel movements as a side effect of the therapy, and one person felt more self-determined as a result of the intervention.

Participants in the hydrotherapy group similarly reported mixed experiences.

Some described an improved sensitivity to temperature and a warming effect of the cold water: “So I experienced how this cold water can generate warmth in the body, so that the body warms up, and that was a positive experience with the knee bath, which is why I really enjoyed doing it, both in the morning and in the evening.” (participant 1). Others noted no perceived benefit in terms of symptom relief: “And after 6 weeks, I stopped again with the hydrotherapy and went back to showering cold in the morning and rubbing myself with a massage glove in the morning, which is also a form of stimulation. And that is certainly not wrong, but it’s also not like it (laughing) would somehow help for those with Restless Legs.” (participant 2).

Improved sleep, a general sense of well-being, and increased self-determination were also reported: “Yes, that I could actually sleep a bit better. I have not taken any sleeping pills or anything.” (participant 4). A few individuals noted a general improvement, as described here: “I really applied it very intensively, and it felt very good. I managed to stick with it, yes, really disciplined, and I must say it felt very good.” (4). One person mentioned that the intervention helped her for more self-determination, and another noted that her immune system improved through the intervention.

### Post-intervention management

Several participants in both groups reported adapting the interventions to suit their personal routines. In the acupressure group, one participant explained: „Yes, mainly the ones on the feet, so I would say the ones between the toes, that is definitely the quickest to do, you can do it anywhere, but mainly I need it on the feet, and then the ones below the knee and those on the ankle are very interesting to me.” (participant 11). Interest in exploring other CAM methods was also mentioned. In the hydrotherapy group, some participants indicated that they continued the intervention or planned to reuse it in the future if symptoms reappeared. “Okay, if it gets worse again, then I’ll just continue with the Kneipp hydrotherapy for a quarter or half a year, and I’m simply confident that there will be an impulse in my body.” (participant 3). Others showed interest in combining it with additional therapies: “Well, I’ll keep trying to find something else (laughs), so to speak, when the 6 weeks of hydrotherapy were over, I started again with fascia, with the fascia roller, because before my surgery, I had no restless legs at all and regularly worked through my body with such a fascia roller and felt that, due to this work with the fascia roller, the restless legs had disappeared.” (participant 2).

## Discussion

This qualitative study provides nuanced insights into how individuals with RLS perceive and experience two self-managed, non-pharmacological interventions—hydrotherapy and acupressure. Rather than merely evaluating symptom change, the study highlights how participants made sense of the interventions in relation to their everyday lives, needs, and expectations.

A central finding was the high value participants placed on flexibility, independence, and low-threshold applicability. These features appear to facilitate a sense of autonomy and self-efficacy, which are known to be important drivers for the acceptance and sustainability of self-managed interventions. Particularly in the context of chronic conditions like RLS, where pharmacological treatment is often associated with frustration and side effects, participants appreciated being able to engage in an approach that was perceived as under their own control and easily integrated into daily routines. This suggests that perceived autonomy may be a key mechanism of acceptability in complementary and integrative care.

At the same time, the findings point to structural and instructional barriers that may limit the full realization of these benefits. For example, participants described uncertainties in applying the techniques correctly—especially in the acupressure group—as well as situational barriers such as physical limitations or lack of time. These barriers reveal a tension between the promise of self-managed care and its practical implementation, indicating the clear guidance, accessible information, and individualized support are crucial for ensuring adherence and perceived benefit.

### Differential response to the study interventions

The impact on the severity of the symptoms varied considerably. In contrast, a qualitative study of hydrotherapy in post-menopausal women found that all participants reported perceived benefits, particularly in relation to sleep, pain relief, emotional stability and improved physical function [39]. One possible reason for the different response is the unclear pathophysiology of restless legs syndrome, the frequent association with comorbidities, and different triggering and aggravating factors The complex pathophysiology of RLS is shaped by the interplay of genetic factors, environmental influences, and a broad spectrum of comorbidities and reinforcing mechanisms (44–48). These heterogeneous outcomes may not only be related to the complex and multifactorial pathophysiology of RLS, but also reflect individual variability in response to non-pharmacological treatments, an aspect similarly observed in a recent study on exercise therapy for RLS (49). The problem of response to treatment exists in both pharmacological and non-pharmacological therapy, particularly regarding the difficulty of achieving a stable treatment response and satisfactory symptom reduction (22, 33, 50, 51). Placebo effects in RLS patients also appear to be particularly high (52), which can also stimulate a positive response. Placebo effects in patients with RLS are not only common but also clinically significant. A large meta-analysis of 85 randomized controlled trials found a clinically relevant placebo-induced improvement (53). This effect could be due to the high expectations of patients. In view of this, patient perceptions in studies with subjective results should be interpreted with caution, as they may be significantly influenced by placebo responses.

In addition, specific studies for different population groups and comorbidities are also recommended in order to develop more targeted treatment strategies (54). Another possible explanation for the lack of noticeable symptom improvements in some participants could be the study’s focus on very specific symptoms. The literature highlights similar challenges in studies, particularly regarding the limitations of available scoring methods for accurate diagnosis and precise outcome measurement (46, 55, 56). Recognizing an improvement in these complex overall symptoms is a major challenge for patients and clinicians. The time course, intensity, frequency and variability of symptoms play a central role and must be carefully considered.

To summarize, RLS is often perceived in everyday life through a variety of interrelated symptoms, such as concentration problems, daytime sleepiness, reduced quality of life, depression, anxiety, reduced well-being and increased stress levels (46, 57, 58).

### Mindfulness, body awareness and sleep

In addition to symptom-related effects, other perceived changes, such as improved mindfulness and sleep, were also reported. So far, there are only a few studies that indicate that applications of CAM can promote mindfulness. A qualitative study on acupuncture revealed mind-body effects after acupuncture (59). Research on mind–body interventions for restless legs remains limited (50). Mind–body medicine is an integrative approach that uses the interactions between mind, body and behavior to promote health and well-being. A Meta-Analysis indicates that various mind–body exercises, such as Pilates, yoga, qigong, and tai chi, have a statistically significant effect on reducing sleep disorders (60).

Insomnia is often the main complaint in patients with RLS and the primary reason for seeking medical help (61, 62), even if it is not part of the clinical diagnosis (23). Difficulty falling asleep, difficulty sleeping through the night and waking up early are the most common concomitant symptoms of insomnia in RLS (23).

Among these, improved sleep quality is frequently reported and appears to be a particularly tangible benefit. In both interventions, participants reported improvements in insomnia, which contributes significantly to quality of life. These perceived changes may be especially noticeable because sleep-related improvements are more immediate and easier for patients to recognize than more subtle emotional or physical sensations associated with mindfulness.

### Individualized adaptation of the study interventions

Adapting the intervention to individual needs seems reasonable for various reasons. For example, some participants felt inadequately guided or experienced uncertainties during the implementation of the intervention. Several interviewees expressed doubts about the correct location or pressure of the acupressure points. Furthermore it may be important to adapt the intervention to individual symptom profiles. Patients’ expectations can also play an important role in the individualized adaptation of treatment approaches. A sequential mixed methods study on CAM in people with multiple sclerosis showed, similar to the present study, that the reasons and expectations for using CAM are diverse. Among them were the desire for symptom relief, prevention, holistic support, and active involvement in managing one’s own health (63). Comparable expectations were reported in the qualitative study on hydrotherapy in postmenopausal women (39). Both studies also highlight that knowledge about the potential applications and mechanisms of CAM among chronically ill patients is often limited, an aspect that should be considered in patient-centered care.

For RLS it has been discussed that the choice of medication should be based on the daily time of onset of symptoms and the individual needs of the patient (55, 64). This underscores the principal that therapies should be adapted according to the course, time of onset and severity of RLS. In addition, the symptoms of restless legs syndrome follow a circadian rhythm, which is closely linked to the diurnal fluctuations of various neurotransmitters and biochemical processes in the body (65). Current research therefore explores how these factors should be taken into account in order to adjust the dosage and timing of medication (65). This is very likely to be relevant also for non-drug treatments.

These aspects could play an important role in future studies aiming to adapt interventions and to examine the subjective differences in effect, such as differences in the perceived intensity of symptom relief, the duration of the effect, the individual tolerance or the improvement in general well-being.

As already mentioned under “Response to treatment,” there is an increasing discussion that all existing comorbidities should be treated specifically. Moreover, depending on whether RLS follows an intermittent, refractory, or chronic-persistent course, individualized and targeted treatment strategies may be appropriate (22, 51).

Although the need for individualized care is widely acknowledged and supported by our findings, further research is needed to determine how such approaches can be practically designed and integrated into routine care.

### Practical implications

The perceived everyday practicality of the intervention played a key role in its acceptance and regular use. Participants highlighted the intervention’s short duration and its easy integration into daily routines. Flexibility regarding location was also considered a major advantage that facilitated participation. These positive perceptions contrast with findings from a mixed-methods study involving a stakeholder workshop on sleep disorders in cancer care, which highlighted that complementary and alternative medicine therapies, such as acupuncture, are often perceived as being associated with structural and financial barriers. Specifically, a lack of qualified personnel, insufficient clinical space, and inadequate reimbursement were identified as major obstacles to the implementation of therapist-guided CAM procedures in routine care (66). In terms of acupressure, some participants expressed a need for re-education or practical assistance in correctly applying the techniques, indicating that self-management can reach its limits without sufficient support.

Hydrotherapy, on the other hand, had a stronger influence on the sensation of temperature and the general well-being.

These differences suggest that the choice of suitable therapy should depend on individual preferences as well as specific physiological and practical conditions. This is supported by a sequential mixed-methods study on CAM use in individuals with multiple sclerosis, which also identified a variety of reasons for use. At the same time, it revealed limited and inconsistent knowledge about CAM highlighting the need for targeted education to support informed choices about CAM use (63).

Overall, it can be said that clear, ongoing guidance is essential to support participants in applying the therapy correctly and confidently. Without it, uncertainties may lead to independent modifications that deviate from the intended treatment plan. Continuous support is also crucial to help integrate the therapy into daily routines and to promote long-term treatment success.

### Limitations

This study was based on a sample of 12 participants, which is appropriate for qualitative research and allowed for thematic saturation. While the data were rich and diverse, certain limitations remain concerning the transferability of the findings to other settings. Participants were recruited from the intervention arms of the HYDRAC study and consented voluntarily. The decision to participate was likely influenced by individual interest in the topic, availability, and personal motivation. This self-selection may have led to a predominance of particularly engaged or health-conscious individuals, potentially introducing a selection bias and narrowing the range of perspectives.

Another limitation regarding transferability lies in the sociodemographic composition of the sample. Participants were recruited exclusively in the Berlin area, and the group lacked diversity in terms of ethnicity and educational background. Since socioeconomic and cultural factors can significantly shape access to and perceptions of CAM, this limited heterogeneity may affect the applicability of findings to more diverse populations (67–69).

In terms of credibility, no participant validation (member checking) was conducted. Transcripts and results were not returned to participants for feedback, as we aimed to minimize participant burden following the intervention period. However, this may have limited the opportunity to verify the accuracy and resonance of the interpretations from the participants’ point of view.

Regarding confirmability and dependability, the coding and interpretation of the data were carried out by a single researcher. Although the analytical process was informed by regular, interdisciplinary team discussions, no formal analyst triangulation was performed. This may have restricted the range of interpretive perspectives and is acknowledged as a methodological limitation.

Additionally, the study applied a partially standardized intervention procedure without adjusting for individual factors such as comorbidities, RLS severity, or disease course. In particular, acupressure could potentially benefit from a more tailored approach that considers clinical or life circumstances. No data were collected on the timing of intervention use or on specific symptom characteristics, which limits the contextual interpretation of individual experiences.

The interviews also revealed that some participants experienced the instructions and support materials as insufficient. This lack of clarity may have contributed to uncertainty during application, which could negatively impact motivation and perceived intervention effects. A further limitation is the absence of interviews with participants from the control group, which restricts comparative insights into their experiences and perspectives.

Finally, the exclusive use of telephone interviews had both strengths and limitations. While this format facilitated participation by individuals with limited mobility and allowed for a broader geographical reach, it limited the ability to observe non-verbal communication and may have influenced the depth and quality of the data. The lack of face-to-face contact might also have made it more difficult to build the rapport necessary for open, reflective responses.

## Conclusion

Participants reported varying degrees of RLS symptom improvement from the self-applied interventions. These findings highlight the need for individualized treatments that consider symptom severity, specific complaints, and accompanying symptoms like insomnia. The interview results show that participants particularly appreciated the simple, flexible, and therapist-independent applicability of the interventions, which allowed them to be used not only at home but also in other everyday settings without dependence on medical infrastructure. A practical, quick-to-implement therapy that can be seamlessly integrated into everyday life is therefore of central importance. Overall, the results make it clear that customized and well-managed therapeutic approaches are essential to strengthen adherence motivation and ensure long-term therapeutic success.

## Data Availability

The datasets used and/or analyzed after completion of the current study will be available upon reasonable request from the corresponding author.
