# Supplementary material for: Hydrotherapy and acupressure for restless legs syndrome: results of a qualitative part of a randomized controlled exploratory study
Source: Front Med (Lausanne). 2025 Sep 1;12:1607307. doi: 10.3389/fmed.2025.1607307 (PMC12433941; doi:10.3389/fmed.2025.1607307)
Supplement: Supplementary file 1 [file Data_Sheet_1.pdf]

HYDRAC Study

Patient interview guide

Version 01 from 05.12.2022

The interview takes place after completion of the study participation (duration of the study 12 weeks).

Questions beginning with a number are key questions.

Indented questions beginning with a letter are possible supplementary questions.

Introduction

I am pleased that you have agreed to an interview. Do you have any questions before we begin?

Opening question

1. How are you currently doing with your restless legs syndrome?

Experience of the therapy and changes

2. You participated in our study. How did you experience your treatment with acupressure or hydrotherapy?

a. About the procedure, what did you like about the self-application, what did you dislike?

b. Why?

3. What changes in your symptoms have you noticed?

a. What do you attribute these changes to?

b. What effects, if any, have you noticed on your life and well-being?

c. Has anything else important changed?

d. If not, how do you feel about that? Why do you think no changes occurred?

Start of the study-expectations and quality of life

4. If you think back to the beginning of the study, what expectations and wishes did you have regarding your treatment?

a. If you had any previous experience, what was it?

Study

5. Looking back, how did you feel about taking part in this study?

a. What did you think of the questionnaires?

b. How did you find completing the diaries?

6. Looking back, can you please describe why you took part in the study?

Conclusion

7. Is there anything else important from your point of view that we have not discussed so far?

8 Has your social environment noticed any changes in you? If so, which ones?

9. Do you have any questions you'd like to ask me?

Thank you very much for the interview!

Basic data

Age:

Gender:

Occupation:

Duration of illness in years:

Subjective severity: mild, moderate or severe?
